# The rate of missed diagnosis of lower-limb DVT by ultrasound amounts to 50% or so in patients without symptoms of DVT: A meta-analysis: Erratum

**DOI:** 10.1097/MD.0000000000018469

**Published:** 2019-12-10

In the article, “The rate of missed diagnosis of lower-limb DVT by ultrasound amounts to 50% or so in patients without symptoms of DVT: A meta-analysis”,^[1]^ which appears in Volume 98, Issue 37 of *Medicine*, the authors need to correct Figure 7 and the related discussion in section 3.7. The corrected section and figure are below.

**Figure 7:**
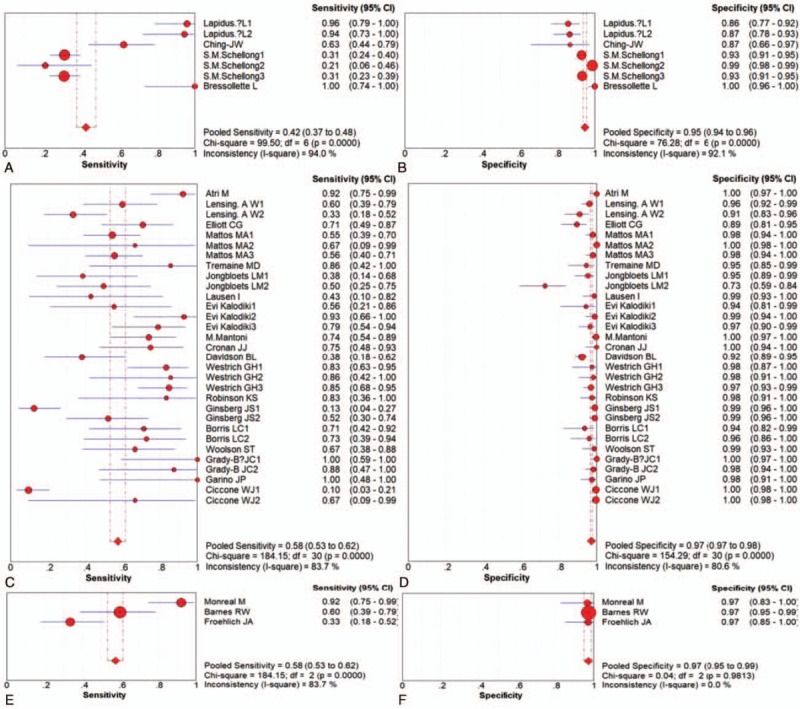
The subgroup analyses of 2000s, 1990 s and 1980 s for DVT.

*3.7. The subgroup analyses of 2000s*, *1990 s and 1980 s for DVT*

Seven individual studies of 2000 s that provided available DATA were included in the quantitative meta-analysis. Subgroup analysis showed that 2000 s for DVT had a poor sensitivity of 42% (95% CI = 37–48%; Fig. 7A) with significant heterogeneity (*I*^2^ = 94.0%, *p* = 0.00), and a higher specificity of 95% (95% CI = 94–96%; Fig. 7B) with significant heterogeneity (*I*^2^ = 92.1%, *p* **=** 0.00).

Thirty-one individual studies of 1990 s that provided available DATA were included in the quantitative meta-analysis. Subgroup analysis showed that 1990 s for DVT had a moderate sensitivity of 58% (95% CI = 53–62%); Fig. 7C) with heterogeneity (*I*^2^ = 83.7%, *p* = 0.00), and a higher specificity of 97% (95% CI = 97–98%; Fig. 7D) with significant heterogeneity (*I*^2^ = 80.6%, *p* **=** 0.00).

Three individual studies of 1980 s that provided available DATA were included in the quantitative meta-analysis. Subgroup analysis showed that 1980 s for DVT had a moderate sensitivity of 0.58% (95% CI = 53–62%; Fig. 7E) with heterogeneity (*I*^2^ = 83.7%, *p* = 0.00), and a higher specificity of 97% (95% CI = 95–99%; Fig. 7F) with heterogeneity (*I*^2^ = 0.0%, *p* *=* 0.98).
